# Review of meningitis surveillance data, upper West Region, Ghana 2009-2013

**DOI:** 10.11604/pamj.supp.2016.25.1.6180

**Published:** 2016-10-01

**Authors:** Robert Domo Nuoh, Kofi Mensah Nyarko, Priscilla Nortey, Samuel Oko Sackey, Noora Charles Lwanga, Donne Kofi Ameme, Culbert Nuolabong, Marijanatu Abdulai, Fredrick Wurapa, Edwin Afari

**Affiliations:** 1 Ghana Field Epidemiology and Laboratory Training Program, School of Public Health, University of Ghana, Legon, Accra; 2 Ghana Health Service

**Keywords:** Meningitis, surveillance, data, Ghana

## Abstract

**Introduction:**

The Upper West region of Ghana is within the meningitis belt. Analysis of long term surveillance data is necessary for understanding changes in the disease occurrence. We analyzed five years of surveillance data to describe by person, place and time and to determine trends in meningitis.

**Methods:**

Meningitis surveillance data from Ghana Health Service in the Upper West Region, from 2009 to 2013 were reviewed. Data was obtained from District-Health Information Management System and line list from the Disease Control Unit. Population figures (denominators) and rainfall data were also analyzed.

**Results:**

Within the period 980 cases of meningitis were reported in the region, 507(52%) females and 473(48%) males. The mean age of cases was 20.1years and standard deviation 18.8 years with, 77.6 %( 761/980) cases occurring in persons aged under 30 years. Children under five years were 19.3% (190/980). Attack rates ranged from 6.1/100,000 population in the Daffiama-bussei-Issa-district to 47.5/100,000 in Jirapa. Overall case fatality rate of meningitis was 12.2% with 14deaths/100,000 population. Bacterial agents were isolated from 35% (245/702) of CSF. Majority were Streptococcus pneumonia 48.2 % ( 122/258), and N. meningitides Y/W 135 40.3% (102/258). Meningitis was found to be seasonal with peaks in the dry season.

**Conclusion:**

Meningitis in the region is seasonal, and showed a decreasing trend. Jirapa, Lawra, Nadowli and Wa West districts had the highest burden. Control effort of the disease should focus on vaccination against streptococcus pneumonia and N. meningitis W135 especially within crowded settlements such as boarding schools.

## Introduction

Meningitis is an epidemic prone disease affecting a significant proportion of the world's population. Case fatality rates may be as high as 50% in the absence of treatment[1]. It is caused by a wide range of agents including bacteria, parasites, viruses, fungi, toxins and even drugs and trauma[1]. Bacterial meningitis is most common form of meningitis and the type called specifically as Neisseria meningitides species is referred to as meningococcal meningitis. Haemophilus influenzae (type b, Hib), Streptococcus pneumoniae, Neisseria meningitidis and Group B Streptococci are common agents of bacterial meningitis. Meningitis is characterized by symptoms ranging from fever, headache, neck stiffness, photophobia, and muscle pains to vomiting and diarrhea. Long term effects of meningitis including loss of hearing, brain damage, learning disability and seizures have been reported among Ghanaian patients[2, 3]. Transmission of the disease is mainly by direct person to person contact, through kissing, air borne in the form droplet infection through sneezing and coughing [4]. About 5%-10% and even up 20% of some populations are carriers of N. meningitis who serve as sources of infection[4–6]. Aside asymptomatic carriage of the disease, dry hot weather and dust also facilitate transmission of the disease while rains reduce the chances of transmission [7]. Factors that can increase a person's risk of bacterial meningitis include: age, community setting, other medical conditions, working with meningitis causative agents and travel to meningitis endemic/epidemic are[8]. Outbreaks of N. meningitides W135 meningitis has been associated with travelers to Mecca during the annual Hajj and Umrah pilgrimage[9]. Case management; early diagnosis and prompt treatment of cases with appropriate antibiotics is recommended. Penicillin, Ceftriaxone Ciprofloxaciline, Rifampicin, and Gentamicin are antibiotics that are commonly used for treatment and as prophylaxis for preventing nosopharyngeal carriage and secondary cases[10, 11]. About 10% of infected patients who receive effective antibiotics still die. Key strategies for control are vaccination, public education and early diagnosis and treatment as well as continues surveillance[4]. Three major vaccine types, conjugate vaccines, polysaccharide Vaccines and Outer membrane protein vaccines have been used successfully for meningitis prevention. conjugate and polysaccharide meningococcal vaccines can be mono valent or tetravalent and are effective and immunogenic among adults and children above 2years[6, 12]. Outer membrane protein (OMP) vaccines are used for vaccination against group B streptococci since scientist failed to develop polysaccharide vaccine for them because antigenic mimicry with human neurologic tissues[13]. In Ghana, meningitides A and C vaccines are routinely used in Ghana[14]. Every year over 400 million people living within the African meningitis belt are at risk of the disease[1]. The African meningitis belt comprising 21 countries and stretching from Ethiopia in East Africa to Senegal in the West bears much of the disease burden [15, 16]. The burden of meningitis however goes beyond this area as far as Namibia and Angola in southern Africa[15]. This changing pattern of the disease has been attributed to climate change[17]. Group A meningococcal meningitis accounts for an estimated 80-85% of all cases in the meningitis belt. This zone has been prone to epidemics occurring at intervals of 7-14 years over the past 100 year[15, 16, 18]. In the 2009 epidemic season, 14 African countries implementing enhanced surveillance, reported 88,199 suspected cases, including 5352 deaths, the largest number since a 1996 epidemic[1]. Ghana recorded a major outbreak in 1997 in which 18,703 cases and 1356 deaths were reported in the three northern regions[19]. The country has also experience outbreaks almost every year in the past five years notably in 2010,2011 and 2013[20–22]. Though only a few of these outbreaks involved large geographical areas the case fatality associated with them was significant (30%-50%)[21]. Meningitis is a priority disease for which continuous surveillance is required in Ghana. Routine data analysis is often limited to short term surveillance data collected within six to twelve month periods. This kind of analysis is limited in value since it does not point out changes in the disease and there is the need to analyze long-term surveillance data for a better understanding of the dynamics of the disease. It continues to be a major public health problem in Ghana and poses significant economic burden to households of infected persons. It is estimated a household in northern Ghana with a non insured meningitis cases looses a little over US 100 dollars while those with patient with health insurance lose about 26 US dollars as meningitis management related cost [23]. Analysis of long-term meningitis surveillance data is necessary for understanding the dynamics of the disease and improving control strategies. We analyzed meningitis surveillance data for the period January 2009 to December 2013 from the Upper West region of Ghana to, understand changes in the disease pattern and make recommendations to improve control measures

## Methods

**Study Design and Data collection:** we reviewed meningitis surveillance data for the period 2009 to 2013 in the Upper West region. Data for reported cases of meningitis was obtained from District Health Information Management Systems (DHIMS 2) and Meningitis case based forms (line list) from the Disease Control Unit of the Upper West Regional Health Directorate. We also obtained Projected Regional and District population figures from the regional disease control office and rainfall figures from the Ghana Meteorological Service for the same period.

**Study site:** the record review covered the Upper West Region of Ghana, which had a projected population of 742,895 in 2013. It is located in northwest Ghana (longitude 1o 25” W and 2o 45” and latitudes 9o 30” N and 11oN). The region experiences a short raining season period from June to October, followed by a long hot dry season from November to May. Maximum temperature is 22Oc in the raining season and 40oC in the dry season. Humidity is typically between 70% to 90% in the raining season but can fall up to 20% in the dry season [24].

**Data Analysis:** Data from case based forms were first entered into excel to create a line list. Case based form data could not be obtained for the year 2009. Data from the District Health Information Management System (DHIMS) database for 2009 was combined with data from the case based forms from 2010 to 2013 to form the final data set. We analyzed data to generate frequencies, trends, geographical distribution, as well as attack rates and case fatality rates. Alert and epidemic thresholds were determined based on WHO guidelines [25]. Data was analyzed using Microsoft excel 2007 and Stata 11.

## Results

**Characteristics of meningitis cases:** within the 5-year period 980 cases of meningitis were reported in the nine districts of the region. Cases were fairly distributed between males and females that is 507(52%) females and 473 (42%) males. The mean age of cases was 20.1 years (SD +/-18.8.) with 77.3 %( 758/980) of cases occurring in persons under 30 years. Children under five years were 19.3% (190/980) Table 1. One hundred and three (103) meningitis deaths were reported in the region, representing an overall case fatality rate of 12.2%. Average attack rate of meningitis for the region during the period was highest in 2010 (85.4 per 100 000) and lowest in 2013 (9.3 per 100 000) Table 2.

**Table 1:** Demographic characteristic of Meningitis Cases reported from the upper west region, Ghana -2009-2010

Characteristic	Total Cases(% of total) n=980 (%)	Cases (2010-2013) n=838	Number of Deaths(2010-2013)	Case Fatality Rate CFR(%)
Sex				
Male	473 (48)	392	54	13.7
Females	507 (52)	507	49	11.0
Age groups				
<5	190 (19.4)	183	10	5.5
5-9	134 (13.6)	125	16	13.3
10-14	130 (13.3)	106	12	11.3
15-19	127 (12.9)	102	8	7.8
20-34	180 (18.3)	150	27	18.0
35-49	129 (13.1)	96	15	15.6
50-59	42 (4.2)	32	5	16.1
60-69	17 (4.2)	16	3	18.7
70+	31 (3.1)	28	7	25.0

**Table 2:** Attack Rates of meningitis by districts, Upper West Region, Ghana-2009-2013. Attack rates/100,000 (AR), AAR(Average Attack for Five years)

	2009	2010	2011	2012	2013	5year Average
Districts	AR	AR	AR	AR	AA	AAR
Jirapa	6.2	143.6	29.9	25.1	29.9	47.5
Lambussie	20.3	58.1	18.9	14.9	14.6	25.4
Lawra	9.9	84.2	29.1	31.5	17.8	33.3
Nadowli	4.1	63.6	33.3	16.3	5.9	24.1
Sisala East	24.7	30.1	36.5	52.8	3.2	24.5
Sisala West	4.3	24.2	47.5	25.3	15.7	21.3
Wa East	0	9.7	10.8	106	3.9	7.3
Wa Municipal	9.5	23.3	115.3	19.8	0.8	15.4
Wa West	100.9	40.6	13.3	14.2	5.8	35.2
Dafiama Bussei	N/A	N/A	N/A	N/A	6.1	6.1
Nandom	N/A	N/A	N/A	N/A	16.3	16.3

Note; Districts where N/A (not applicable) is indicated were created after 2012.

**Laboratory findings:** lumber puncture was performed in 85.1% (715/840) of the cases between 2010 and 2013. All samples were tested by standard laboratory techniques, including serology, gram staining and culture. Organisms were isolated from only two hundred and fifty three (253) representing 38.8% of cases tested. Streptococcus pneumonia and N. meningitides Y/W 135 constituted almost 90% of the organisms isolated with 48.2% and 40.3% respectively. Other isolates were N. meningitides A (2.7%), N. meningitides B (1.2%), group B streptococcus 3.6%, Hemophilus influenza (2.4%) and N. meningitides C (1.6 %.). Out all cases tested, 60.8% produced a negative result. About 38% (278/715) of cases were confirmed by laboratory test. Twenty-five cases were reported only as positive or based on gram reaction without specifying the type of organism Table 3.

**Table 3:** CSF (cerebrospinal fluid) laboratory test results of Meningitis cases, Upper West Region Ghana. 2010-2013

Number CSF collected for testing: 85.1% all cases (715/840)	
	N=715 (%)
Number positive by at least one lab technique	278(38.8%)
Number Negative by at least one lab technique	435 (60.8%)
Number positive With organism clearly identified by culture/serology/PCR	253 (35.4%)
Number positive (reported only as gram positive cocci)	20(2.8%)
Number positive (reported only as gram negative cocci)	5 (0.7%)
Insufficient CSF specimen for testing	2(0.3%)
Characteristics of Bacterial Agents Isolated	N=253 (%)
Streptococcus pneumonia	122(48.2)
Neisseria meningitides Y/W135	102(40.3%)
Group B streptococci	9 (3.6)
Neisseria meningitides A.	7 (2.7)
Hemophilus influenzae	6 ((2.4)
Neisseria meningitides C	4 (1.6)
Neisseria meningitides B.	3(1.2%)

**Figure 1:**
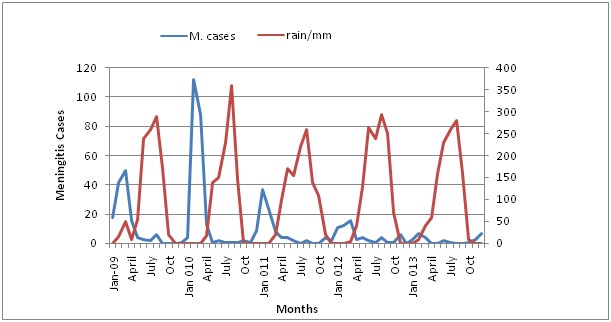
Meningitis Cases and rainfall trends in Upper West Region Ghana, 2009-2013

**Time Trends of cases:** meningitis cases in the region demonstrate strong seasonality with most peaks occurring between January to April each year during the dry weather. Cases begin to decline by May each year with the onset of the rains Figure 1. In 2010 the following outbreaks occurred, Jirapa in week 6(13 cases), week 7(29cases), week 8(18cases), Lawra week 11(9cases) Sisala East, and Wa West. Based on the same threshold the outbreaks were recorded after 2011, however sporadic cases of meningitis occurred in some districts.

**Geographic trends of meningitis cases:** four districts, Jirapa, Lawra, Nadowli and Wa West account for over 65% (636/980) of all reported cases of meningitis in the region from January 2009 to December 2013. The worst affected district was Jirapa with 21.5% of all cases (212/980). It had the highest attack rate of 143/100,000 population in 2010. The average attack rate over the 5-year period was however 47 people/ 100,000 population. Wa East was the least affected district with 2.7% all cases and an average attack rate of 7/100,000 population Table 2.

## Discussion

Meningitis remains a public health concern in Ghana over 3 million people at risk each year[14]. Emerging strain Neisseria meningitides and changing environmental conditions could change the patterns of epidemic meningitis in Ghana. Internationally acceptable case fatality is 10% with most countries in sub Saharan Africa going beyond this level. Typically meningitis case fatalities in Ghana are often above this threshold[14, 21]. This could be due to the level of preparedness and response in the district within the meningitis belt in Ghana Meningitis cases in the region showed strong seasonality, peaking each year in March during the dry season and declining to the lowest in the peak of the raining season. There was a declining trend of meningitis cases in the region over the years. This could be attributed to a mass vaccination campaign in the region after the 2010 epidemic[26, 27]. Similar results have also been observed following mass vaccination campaigns in Mali, Burkina Faso and Niger [28]. Meningitis cases in the region was higher among younger age groups and adults below 35 years. This could be due to the increased likelihood of this group of people participating in activities in overcrowded places like schools, markets and other work places. Based on thresholds set using WHO guidelines[25], some Districts recorded outbreaks during the period. Outbreaks occurred in Jirapa, Lawra, Lambussie, and WaWest, Nadowli, Sisala East and Wa East Districts. In 2010 the following outbreaks occurred in Jirapa between weeks 6 and 10 in which over 80 cases were reported. Lawra Wa East, Nadowli and Wa West also had outbreaks in week 11 of the same year. The Jirapa district recorded the highest number of cases, over 200 within the period, and also recording an attack rate of 144.7/100,000 in 2010. N. meningitides Y/W 135 and streptococcus pneumonia were the most predominant isolates associated with meningitis outbreaks in the region contributing about 40% and 49% of all isolates respectively.

This finding of this study agrees with that of other researchers who observed a similar increase in incidence of pneumococcal meningitis in Ghana and other African countries[29]. N. meningitides W135 serogroup which was once rare in Ghana, with only four cases reported in 2004 has now become a common cause of outbreaks the region[3] This suggest that if control and prevention efforts need to be improved, mass vaccination campaigns must target N. meningitis, W135 and streptococcus pneumonia which are the most common agents isolated from the region. Contrary to previous findings that 80 -85% of all of meningococcal meningitis in the African Meningitis belt, are due to sero-group A[27, 1] a different trend is observed in this study where N. meningitis Y/W 135 constitute up to 40% of all isolates of bacterial meningitis from outbreaks in Ghana. This emerging trend in which N. meningitidis is increasingly becoming major cause of bacterial meningitis in the upper west region is probably due to the region's proximity to Burkina Faso, which has recorded several outbreaks of W135. It therefore implies that meningitis vaccines used for prevention should include antigen that could prevent W135 strains as currently the vaccines in use are against sero-group A and C. Meningitis cases were generally high in 2010 across all district, however case fatality for most districts was below or within the 10.6% Within the WHO acceptable case fatality rate. Children under five years old contributed almost 20% of meningitis case, however, they had the least case fatality. This suggest that public health intervention to promote child health through routine vaccinations efforts[14, 21]. In 2011, cases reduced remarkably with attack rates decreasing for all districts, but case fatality rates were far above WHO standards for most districts. Though fewer cases are reported among the elderly (70 year and above) case fatality rate appear to be higher among them. This suggests that case management during 2011 was probably not very effective. Similarly the Wa East district consistently recorded fewer cases than other districts but maintained a high case fatality ratio. The use of secondary data presented some limitations. First, data obtained from the DHIMS data base was in predetermined age groups which influenced the selection of final age categories used in this analysis. Also mortality data for 2009 could not be obtained.

## Conclusion

The analysis demonstrates a consistent decline in meningitis cases over the years, though, Streptococcus pneumonia and N. meningitides Y/W135 were major isolates of all laboratory confirmed cases. The disease showed strong seasonality and mostly persons under 30 years were involved. Case fatality of meningitis was generally higher than the WHO accepted level and was even higher among those aged seventy years or more. In conclusions, meningitis data collected in the region is relevant for preventing meningitis outbreaks in the region and improving control measures, since findings from analyzing this data could influence critical decisions like selecting vaccines for the population. This data is also useful in evaluating the impact of public health interventions.

**Recommendations:** we recommend that health authorities should focus vaccination effort towards N. meningitides Y/W 135 and Streptococcus pneumonia as this have been found to be the predominant circulating strain. Polyvalent vaccine targeting several causative agents of meningitis would be the best option to consider for preventing these agents.
